# Usefulness of Thoraco-Abdominal Synchrony Assessment in Hospitalized COPD Exacerbations Using Respiratory Inductance Plethysmography—A Pilot Study

**DOI:** 10.3390/jcm15051942

**Published:** 2026-03-04

**Authors:** Mara Santomassimo, Cristina Lalmolda, Berta Lloret, Inés Ruiz, Manel Lujan

**Affiliations:** 1 Pneumology Service, ASST Valle Olona-Busto Arsizio, 21052 Busto Arsizio, Italy; santomassimo.mara@gmail.com; 2 Servei de Pneumologia, Parc Taulí Hospital Universitari, Institut d’Investigació i Innovació Parc Taulí (I3PT-CERCA), Universitat Autònoma de Barcelona, 08208 Sabadell, Spain; clalmolda@tauli.cat (C.L.); blloret@tauli.cat (B.L.); iruiz@tauli.cat (I.R.)

**Keywords:** asynchrony, global phase delay, phase angle

## Abstract

**Background/Objectives:** Thoraco-abdominal asynchrony (TAA) is a key mechanical consequence of severe chronic obstructive pulmonary disease (COPD), particularly during acute exacerbations (AECOPD), when dynamic hyperinflation and diaphragmatic dysfunction impair the coordination between rib cage and abdominal motion. Continuous, non-invasive monitoring of respiratory mechanics may provide valuable information on clinical evolution during hospitalization. This study aimed to evaluate Global Phase Delay (GPD) as a longitudinal marker of TAA in hospitalized AECOPD patients and to explore its ability to reflect disease severity and short-term clinical evolution using repeated measurements obtained with thoracic and abdominal respiratory belts using respiratory inductance plethysmography (RIP). **Methods:** We conducted an observational longitudinal study in hospitalized adults with AECOPD. Respiratory inductance plethysmography signals were recorded daily over four consecutive days using thoracic and abdominal RIP belts. Five-breath sequences were analyzed to derive GPD, phase angle, and loop rotation direction through automated MATLAB processing. Clinical data included demographics, lung function, blood gases, dyspnea severity, and need for intermediate respiratory care unit (IRCU) admission. Temporal changes in TAA indices and subgroup differences (FEV_1_ < 35%, IRCU admission) were assessed using repeated-measures ANOVA. **Results:** Twenty-one patients were included. On admission, mean absolute GPD was 49 ± 58°, with larger delays observed in patients with more severe airflow limitation and in those requiring IRCU support. During hospitalization, GPD showed a significant reduction over time (*p* < 0.05), particularly in these subgroups, indicating progressive improvement in thoraco-abdominal synchrony. Directional analysis of GPD revealed heterogeneous patterns consistent with different underlying mechanical behaviors. **Conclusions:** Serial assessment of TAA using respiratory bands and GPD provides clinically meaningful information on the evolution of respiratory mechanics during AECOPD hospitalization. This approach may support bedside monitoring and help track patient response to treatment, offering potential value for individualized respiratory management.

## 1. Introduction

Chronic obstructive pulmonary disease (COPD) is a leading cause of global morbidity and mortality, characterized by persistent airflow obstruction and significant alterations in respiratory mechanics. This pathological condition results from chronic airway inflammation, leading to progressive narrowing of the bronchial lumen, destruction of pulmonary parenchyma (emphysema), and dynamic hyperinflation. These pathological processes impair ventilation-perfusion matching and increase the work of breathing, directly affecting thoraco-abdominal movements [1,2,3].

In healthy individuals, thoraco-abdominal movements are synchronous, with the rib cage and abdomen working in harmony to optimize ventilation. In COPD patients, however, this normal synchrony is often disrupted, giving rise to thoraco-abdominal asynchrony (TAA). Chronic pulmonary hyperinflation significantly contributes to diaphragmatic dysfunction, reducing its excursion and altering its functional geometry [4,5]. Under severe hyperinflation, the diaphragm flattens, losing part of its contractile ability and transferring a greater portion of the respiratory workload to accessory muscles [6]. Additionally, rib cage stiffness further limits thoracic expansion, resulting in mechanical inefficiency and worsened overall ventilation [1,5,6,7].

For COPD patients, TAA manifests as increased dyspnea perception and significantly impacts daily life through elevated energy expenditure during breathing, accelerated muscle fatigue, and compromised quality of life [4,6,8].

The quantitative assessment of thoraco-abdominal movements has evolved significantly since Konno and Mead’s pioneering work [3], establishing the foundation for modern asynchrony measurement techniques. Respiratory inductance plethysmography (RIP) emerged as a transformative technology, offering enhanced capabilities for monitoring compartmental synergy across diverse clinical scenarios. Recent research has improved understanding of thoraco-abdominal belt applications in COPD management, though significant challenges remain in clinical implementation [7,8,9].

**Figure 1:**
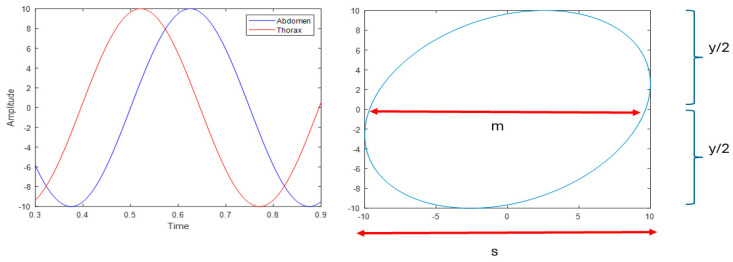
Native tracing (amplitude vs. time), corresponding Lissajous plot, and thoracoabdominal asynchrony (TAA) quantified by the phase angle. The phase angle was calculated from an automatically generated sequence with a 75° phase shift and defined as sin^−1^(m/s).

Lissajous figures represent a graphical method for analyzing the relationship between thoracic and abdominal movements during respiration. The phase angle, derived from these figures, quantifies the degree of asynchrony between thoracic and abdominal movements during the respiratory cycle [3,10]. A small phase angle indicates near-synchronous movements, while larger values reflect increasing asynchrony. However, due to mathematical constraints, the calculated phase angle cannot exceed 90°, even in cases of complete paradoxical breathing [11]. Figure 1 shows the calculation of the phase angle in a sequence generated automatically with a 75-degree phase shift.

Despite their clinical utility, Lissajous figures and phase angle calculations present several notable methodological limitations, including estimation based on a single point measurement, mathematical constraints limiting values to 0–90 degrees, and directional ambiguity regarding which compartment leads the respiratory cycle.

**Figure 2:**
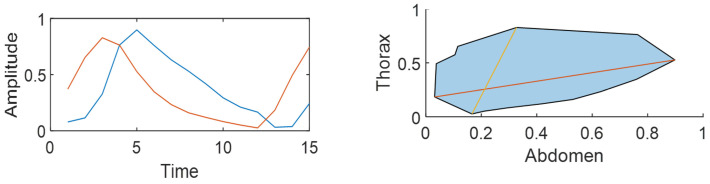
Example of global phase delay (GPD) estimation in a clinical sample. The thoracic belt leads (red line), resulting in a positive GPD (clockwise rotation). In this example, GPD is 57.05 degrees, indicating moderate thoracoabdominal asynchrony (TAA).

To overcome these limitations, the global phase delay (GPD) has been proposed as a comprehensive and cumulative measure of phase shift observed across an entire respiratory cycle. Based on the ratio of vectors representing both belts, GPD is calculated from the slopes of both vectors and assigned a positive sign if the thoracic belt is advanced and negative if the abdominal belt leads [12]. Recent studies have demonstrated that GPD covers the whole spectrum of thoraco-abdominal asynchrony situations in a single value, eliminating drawbacks associated with classical phase angle measurement [13]. Figure 2 shows an example of the estimation of global phase delay in a clinical sample with advanced thorax and a GPD value of 57.05 degrees.

This study investigates the relationship between respiratory movement patterns and clinical outcomes in acute exacerbations of COPD (AECOPD). The central hypothesis is that thoraco-abdominal synchrony, quantified by GPD measurements, reflects aspects of the clinical course of AECOPD. The primary objective is to perform a longitudinal assessment of GPD as an indicator of thoraco-abdominal asynchrony in patients hospitalized for AECOPD. Secondary objectives include exploring associations between GPD measurements and clinical parameters, such as dyspnea severity and arterial blood gas values, during hospitalization. All analyses are exploratory and aim to provide physiological insights rather than predictive clinical conclusions.

## 2. Materials and Methods

### 2.1. Design

An observational and longitudinal study was designed to analyze thoraco-abdominal asynchrony in patients hospitalized for acute exacerbations of chronic obstructive pulmonary disease (AECOPD). This study was designed as a proof-of-concept physiological study. No formal sample size calculation was performed, as no predefined effect size or clinical endpoint was targeted. Upon obtaining informed consent, all personal and biographical data were collected and documented in a dedicated case report form (CRF). Each participant was assigned a unique alphanumeric code to ensure anonymity through pseudonymization, with additional information stored separately and safeguarded by technical and organizational measures.

### 2.2. Inclusion and Exclusion Criteria

Patients were eligible for inclusion if they had confirmed COPD diagnosis, defined by a smoking history exceeding 20 pack-years and spirometry demonstrating FEV_1_/FVC ratio below 70%. Additionally, patients required hospitalization for acute COPD exacerbation and met at least one of three clinical criteria: moderate-to-severe dyspnea with signs of increased work of breathing, including use of accessory muscles or paradoxical abdominal movement; requirement of FiO_2_ exceeding 0.4 to achieve adequate oxygenation (88–92% in patients at risk of hypercapnia, or greater than 92% in other patients); or acute ventilatory failure characterized by pH below 7.35 with PaCO_2_ exceeding 45 mm Hg. Patients with lung consolidation, acute heart failure, or high clinical suspicion of pulmonary embolism were excluded because these conditions may independently alter respiratory mechanics and gas exchange, thereby confounding the interpretation of monitoring-derived variables. These exclusions were deliberately applied to minimize physiological confounding and to allow a more accurate interpretation of thoracoabdominal interaction patterns in AECOPD.

### 2.3. Signal Acquisition

Respiratory tracings were obtained using two inductive belts (Z-RiP, Philips Respironics, Murrysville, PA, USA) positioned around the thorax (upper chest, below the armpits and above the nipples) and the abdomen (at the waistline) and connected to an analog-to-digital converter (BIOPAC MP150, Goleta, CA, USA). Signals were acquired at a sampling frequency of 500 Hz, with a band-pass filter (0.05–2 Hz) applied prior to analysis. Measurements were performed during spontaneous breathing with the fraction of inspired oxygen (FiO_2_) adjusted to maintain oxygen saturation (SpO_2_) between 88% and 92% for 10 min, in the supine position with a maximum trunk incline of 30°. Daily recordings included simultaneous capnography traces.

Sequences of five breaths were selected daily from belt recordings, and automated analyses computed phase angle, loop rotation (clockwise or counterclockwise), global phase delay (GPD), and loop area using a MATLAB v.2022 script (protected under Safe Creative 2404247746604-2UC6ZP). Briefly, the system automatically identifies ventilatory cycles based on the detection of local maxima and minima, determines the dominant band according to its degree of phase shift, and calculates the GPD as described elsewhere [12].

### 2.4. Data Collection

Comprehensive data collection included demographic characteristics, anthropometric measurements, smoking habits, disease severity markers (respiratory function tests, home therapies, exacerbation frequency, CAT score), characteristics of the present exacerbation (evolution days, bronchospasm, fever, consciousness level, chest X-ray findings, admission arterial blood gas values, respiratory monitoring unit admission need), and daily evolution parameters (phase angle, GPD, SpO_2_, transcutaneous CO_2_, respiratory rate).

### 2.5. Statistics

Statistical analysis employed the Shapiro–Wilk test to evaluate normality of distribution. Normally distributed variables were reported as mean and standard deviation, while non-normal distributions were presented as median and interquartile range. Student’s *t*-test for unpaired data compared groups based on thoracoabdominal synchrony, and mixed ANOVA for repeated measures assessed evolution of measurements across different days. When the sphericity of the model could not be assumed (Mauchly’s test with *p*-value < 0.05), the significance of the model was assessed with the Greenhouse–Geisser correction. Statistical significance was set at *p* < 0.05, with all analyses performed using SPSS version 25.

## 3. Results

### 3.1. Clinical, Analytic and Anthropometric Data

A total of 21 patients (10 males) were monitored during the first four days of hospitalization. Anthropometric characteristics, pulmonary function data, chronic home therapies, and history of exacerbations in previous years are summarized in Table 1. The mean age was 64 ± 7.18 years, mean BMI was 23.95 ± 6.46 kg/m^2^, mean FEV_1_ was 0.98 ± 0.43 L (36.3 ± 12.5% of predicted), mean FVC was 2.75 ± 0.77 L (74.05 ± 19.9% of predicted), and the mean COPD Assessment Test (CAT) score was 22.23 ± 11.05. Seven patients had an FEV_1_ below 35% of predicted.

Data at hospital admission are presented in Table 2. Nine patients required admission to the respiratory monitoring unit with non-invasive ventilatory support. All patients were discharged following resolution of the exacerbation that led to hospitalization. The mean length of hospital stay was 17.2 ± 12.3 days.

**Figure 3:**
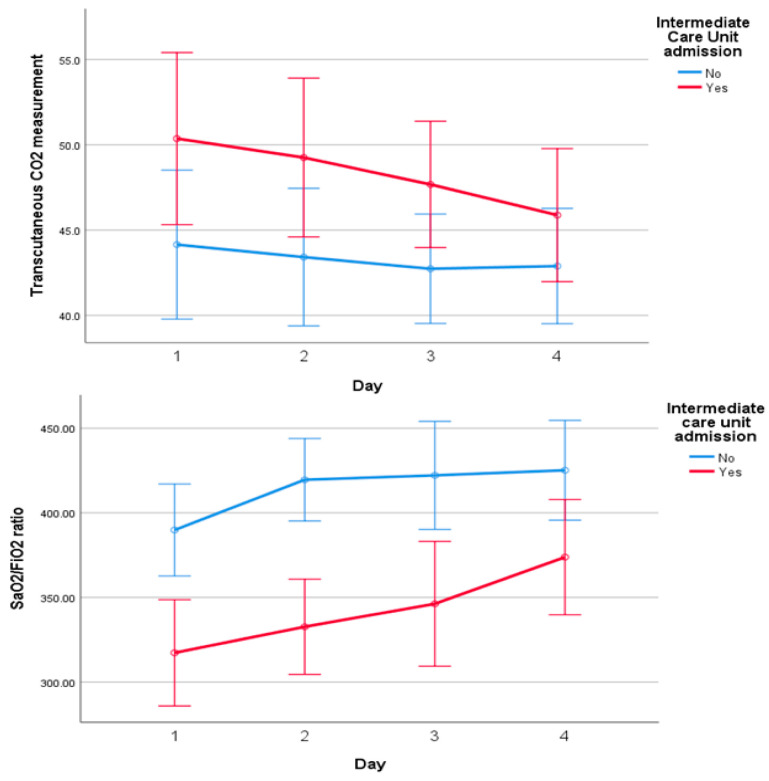
Evolution of transcutaneous CO_2_ and SpO_2_/FiO_2_ ratio during the first four days of hospitalization, stratified by admission to the intermediate care unit. SpO_2_/FiO_2_ increased significantly both within and between groups, whereas the decrease in transcutaneous CO_2_ reached borderline statistical significance.

All patients underwent daily transcutaneous CO_2_ monitoring. Figure 3 shows the evolution of transcutaneous CO_2_ levels over the four days of data collection, together with the SpO_2_/FiO_2_ ratio. The increase in SpO_2_/FiO_2_ was significant both within and between groups (*p* < 0.05 ANOVA test for repeated measures, Eta-squared 0.474), while the decrease in transcutaneous CO_2_ was at the limit of statistical significance, with a more pronounced trend in patients admitted to the intermediate care unit (*p* = 0.06, Mauchly W test 0.323, Eta-squared 0.165). Regarding respiratory rate (RR), a slight non-significant decrease was observed over the first four days of admission. Interestingly, likely because of non-invasive ventilation, patients in the intermediate care unit did not exhibit higher respiratory rates than those admitted directly to the ward.

### 3.2. Plethysmographic Data

The absolute mean phase lag upon admission, measured by GPD, was 49 ± 58 degrees. Notably, only two patients exhibited severe counterclockwise rotation upon admission (abdomen leading: −69 and −161 degrees), while four patients showed severe clockwise rotation (thorax leading) with abdominal paradox (with a mean GPD value of 123 degrees). No correlation was observed between baseline GPD and CAT score, transcutaneous CO_2_, or SpO_2_/FiO_2_ ratio.

Table 3 shows GPD values (considering the sign), absolute GPD values, and phase angle during the first day of monitoring based on severity of obstruction and need for admission to the Intensive Respiratory Care Unit (IRCU). Patients with very severe COPD (FEV_1_ < 35%) exhibited significantly greater absolute deviations upon admission compared to those with FEV_1_ > 35% (85.0 ± 65.7 vs. 31.82 ± 46.82 degrees, *p* < 0.05). Similarly, patients requiring IRCU admission showed higher absolute GPD values compared to those not requiring IRCU (75.48 ± 63.15 vs. 30.13 ± 47.37 degrees, *p* = 0.06). Phase angle measurements revealed significant differences only between IRCU and non-IRCU groups (49.8 ± 29.8 vs. 22.36 ± 16.35 degrees, *p* < 0.05), but not between FEV_1_ severity groups.

**Figure 4:**
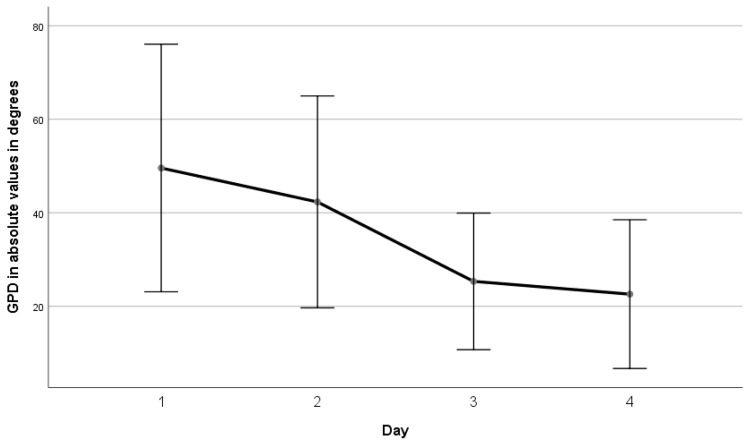
Daily mean absolute values of global phase delay (GPD) during hospitalization. Differences over time were statistically significant (*p* < 0.05, repeated-measures ANOVA).

**Figure 5:**
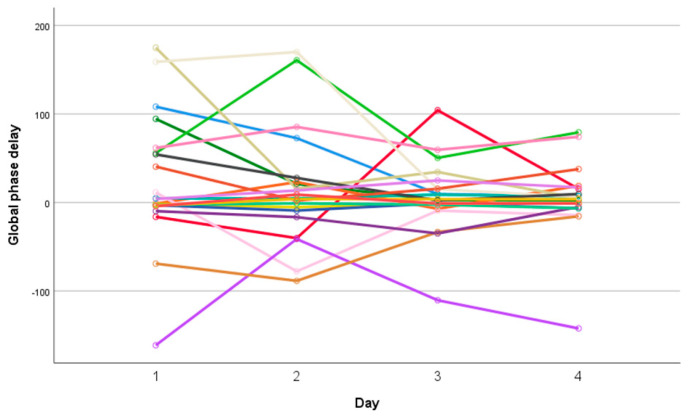
Individual global phase delay (GPD) values for each patient during the first four days of hospitalization.

A significant reduction in absolute GPD values was observed over the monitoring days (*p* < 0.05, repeated measures ANOVA, Eta-squared 0.218), as illustrated in Figure 4. Figure 5 presents individual GPD values for each patient during the first four days of hospitalization, considering the direction of deviation.

**Figure 6:**
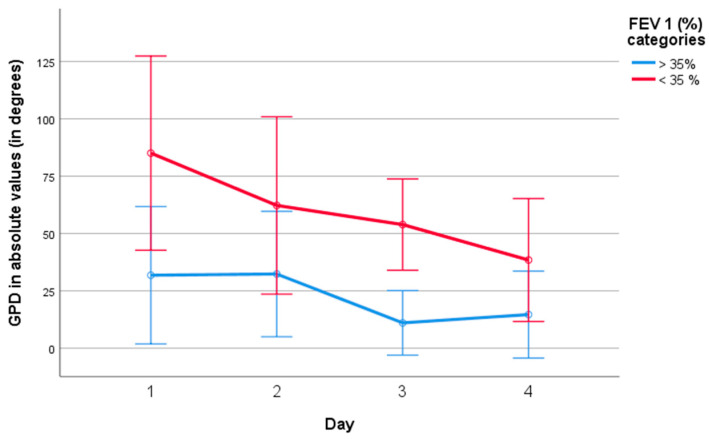
Evolution of absolute global phase delay (GPD) values stratified by FEV_1_ < 35% or ≥35%. Between-group differences were statistically significant (*p* < 0.05, repeated-measures ANOVA).

Differences in the evolution of absolute GPD values were noted between patients with FEV_1_ < 35% and those with higher values, as shown in Figure 6. Patients with FEV_1_ < 35% demonstrated higher initial deviations that progressively decreased (*p* < 0.05 intergroup, repeated measures ANOVA, Eta-squared 0.323). These differences were not evident when using phase angle measurements.

**Figure 7:**
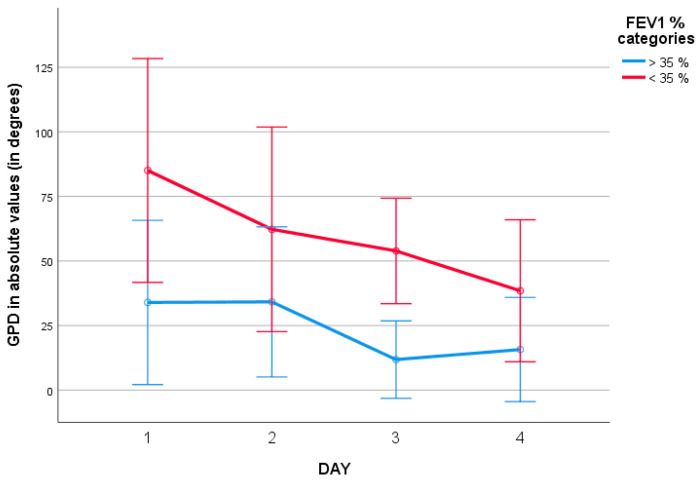
Evolution of absolute global phase delay (GPD) values according to admission to the intermediate respiratory care unit (IRCU). Between-group differences showed a trend toward statistical significance (*p* = 0.06, ANOVA).

A similar trend was observed when comparing patients requiring IRCU admission with those who did not (*p* = 0.09, repeated measures ANOVA, Eta squared 0.0123, Figure 7). In the intra-subject analysis, patients admitted to IRCU showed a significant reduction in thoracoabdominal asynchrony during the monitoring period (*p* < 0.05, repeated measures ANOVA), while this improvement was not observed in patients who did not require IRCU admission (*p* = 0.5). These differences were also not apparent when using phase angle measurements.

## 4. Discussion

This study evaluated thoracoabdominal asynchrony (TAA) in patients COPD exacerbation during the first four days of hospitalization, focusing on deviations in Global Phase Delay (GPD) as a marker of respiratory mechanics. The results revealed that patients with severe airflow obstruction and those requiring admission to an intensive respiratory care unit (IRCU) exhibited greater absolute GPD deviations upon admission, indicating a higher degree of asynchrony compared to other subgroups. Furthermore, normalization of GPD became more evident over the course of hospitalization in these patients, suggesting that GPD monitoring could be particularly valuable in this subgroup to assess therapeutic response and guide individualized respiratory care.

### 4.1. Evolution of GPD and Its Clinical Implications

During daily monitoring, a significant reduction in absolute GPD deviations was observed over the four days of hospitalization in patients with FEV_1_ < 35% and those admitted to IRCU. This finding highlights the potential role of GPD as a sensitive marker for monitoring improvements in thoracoabdominal synchrony, even in the context of non-invasive ventilatory support. However, the lack of significant differences in phase angle values limits the comparability with other studies, which have used different methodologies for assessing TAA.

The greater GPD deviations observed in more severe patients may be explained by distinct pathophysiological mechanisms. In cases of severe diaphragmatic dysfunction and fatigue, the abdominal paradox—a characteristic inward abdominal motion during inspiration—becomes evident. Excessive lung hyperinflation, whether disease-related or potentially exacerbated by non-invasive mechanical ventilation, may further contribute to diaphragmatic dysfunction by increasing the curvature of the diaphragm and reducing its contractile efficiency. In the absence of direct measurements of lung volumes or diaphragmatic function, it is therefore difficult to determine the predominant mechanism underlying TAA due to diaphragmatic dysfunction in individual patients. A recent article highlights the existence of this dual mechanism in patients during exercise [13]. Conversely, in patients with preserved diaphragmatic function but significant hyperinflation and air trapping, thoracoabdominal dynamics are altered due to delayed thoracic activation and counterclockwise rotational motion, commonly referred to as Hoover’s sign [6].

While the pathophysiological interpretation of the results remains coherent and well-argued, statements regarding the guidance of non-invasive ventilation or individualized patient management have been reformulated as hypotheses or future perspectives. Transient increases in GPD observed over short intervals should be interpreted with caution, as they may reflect heterogeneous mechanical responses during recovery, including secretion retention, fluctuating hyperinflation, NIV intolerance, or changes in respiratory muscle recruitment. These contrasting mechanisms highlight the heterogeneity of thoraco-abdominal asynchrony in severe COPD and support the potential value of GPD for detecting and differentiating these dysfunctions. Monitoring prolonged trends over time may provide additional physiological insights and generate hypotheses for future interventional studies, rather than serving as direct therapeutic guidance

### 4.2. Clinical Relevance

The use of GPD measured via inductive plethysmography offers a non-invasive, reliable method for assessing TAA in patients with severe COPD. This approach may support the optimization of respiratory support settings, particularly in patients with severe clockwise rotation or abdominal paradox. Additionally, identifying these patterns may facilitate the personalization of rehabilitation programs, potentially improving clinical outcomes, especially after acute exacerbations. Our results further suggest that the clinical relevance of monitoring thoracoabdominal asynchrony is likely greatest in patients with severe exacerbations admitted to intermediate care units, as this is the cohort in which we observed the most pronounced changes in TAA over time. These findings highlight the potential utility of prolonged and repeated monitoring to guide therapeutic decisions and to better understand patient-specific recovery trajectories.

### 4.3. Study Limitations

This study has several limitations. First, the small sample size limits statistical power and restricts the generalizability of the findings, particularly for subgroup analyses. Second, the observational design limits causal inference, and the observed changes in thoracoabdominal asynchrony may reflect the natural course of recovery and the effects of standard treatments administered during hospitalization. Ideally, the evolution of thoracoabdominal asynchrony in patients with treatment failure (defined as need for endotracheal intubation, ICU admission, or death) should have been compared with that of patients with favorable clinical evolution; however, no such events occurred in our cohort.

In addition, the impact of different ventilatory support modalities was not systematically evaluated, and the individual pathophysiological mechanisms underlying thoracoabdominal asynchrony were not specifically explored. Finally, the follow-up period was limited to the first days of hospitalization, precluding assessment of longer-term trends and outcomes. Future studies should aim to include larger cohorts and longer follow-up periods to better evaluate the role of GPD as a monitoring tool in patients with severe COPD.

## 5. Conclusions

Patients with acute COPD exacerbations, FEV_1_ < 35%, or requiring admission to respiratory monitoring units exhibit pronounced thoracoabdominal asynchrony, as reflected by Global Phase Delay (GPD).

Inductive plethysmography-based GPD assessment offers a practical method for evaluating thoracoabdominal synchrony in individuals with advanced COPD or those admitted to intensive respiratory care.

Unlike prior research, the present study establishes a relationship between GPD deviations and both clinical and functional characteristics of patients, underscoring the utility of GPD as an objective measure of respiratory impairment severity. Tracking variations of this parameter can enhance understanding of disease progression and treatment outcomes, paving the way for tailored respiratory interventions and improved patient management in the era of personalized medicine. However, these observations must be confirmed in larger studies including broader patient populations.

## Figures and Tables

**Table 1:** Anthropometric characteristics, smoking exposure, lung function and clinical status of the study population. Data are expressed as mean ± standard deviation (SD), unless otherwise stated. Smoking exposure is reported in pack-years. Chronic home respiratory therapies are expressed as percentage of patients.

Parameter	Mean	SD
**Age**	64	7.18
**BMI**	23.95	6.46
**Smoking habits in pack/year (63% smokers, 37% former slokers)**	45	19.5
**FEV_1_ (L)**	0.98	0.43
**FEV_1_ (%)**	36.3	12.5
**FVC (L)**	2.75	0.77
**FVC (%)**	74.05	19.9
**COPD assessment test (CAT)**	22.23	11.05
**Exacerbations previous 2 years (number)**	6	4.81
**Hospital admissions previous 2 years (number)**	3.29	4.37
**Chronic home therapies**	41% oxygen users 22.7% NIV users 4.5% CPAP users.	

**Table 2:** Clinical, respiratory and arterial blood gas findings at hospital admission. Data are expressed as mean ± standard deviation (SD), unless otherwise stated. Clinical and radiological findings are expressed as percentage of patients.

Parameter	Mean	SD
**Onset of symptoms before admission (days)**	4.9	3.2
**Respiratory rate at admission**	18.9	3.8
**Arterial blood gases at admission (Emergency department)**		
**pH** **PaCO_2_ (mmHg)** **PaO_2_ (mm Hg)** **FiO_2_ (%)**	7.35 57.6 81.4 27.7	0.1 17.6 42.4 6.1
**Clinical and radiological findings (%)**		
**Bronchospasm** **Confusion** **Fever (>38.5 °C)**	95% 9% 13%	

**Table 3:** Global phase delay (GPD, signed and absolute values) and phase angle at admission according to lung function severity and need for intermediate respiratory care unit (IRCU) admission. Data are expressed as mean ± standard deviation (SD). Comparisons were performed between patients with FEV_1_ ≥ 35% vs. FEV_1_ < 35%, and between patients admitted or not admitted to the IRCU.

	Global Phase Delay (with Sign)	Global Phase Delay (Absolute)	Phase Angle
FEV_1_ > 35%	29.4 ± 48.4	31.82 ± 46.82	29.83 ± 27.42
FEV_1_ < 35%	11.72 ± 112	85.0 ± 65.7	42.7 ± 23.84
*p* value	0.61	<0.05	ns
Non-IRCU	14.5 ± 54.75	30.13 ± 47.37	22.36 ± 16.35
IRCU	35.30 ± 95.14	75.48 ± 63.15	49.8 ± 29.8
*p* value	0.53	0.06	<0.05

## Data Availability

The data supporting the findings of this study are available from the corresponding author upon reasonable request for academic and research purposes. De-identified data and analysis code can be shared, subject to ethical approval and privacy considerations.

## Abbreviations

The following abbreviations are used in this manuscript:

AECOPD	Acute exacerbation of chronic obstructive pulmonary disease
TAA	Thoraco-abdominal asynchrony
PhAng	Phase Angle
GPD	Global Phase Delay
IRCU	Intensive Respiratory Care Unit
